# A Comprehensive Review on Therapeutic Perspectives of Phytosterols in Insulin Resistance: A Mechanistic Approach

**DOI:** 10.3390/molecules27051595

**Published:** 2022-02-28

**Authors:** Monisha Prasad, Selvaraj Jayaraman, Mohamed Ahmed Eladl, Mohamed El-Sherbiny, Mosaab Abdella Ebrahim Abdelrahman, Vishnu Priya Veeraraghavan, Srinivasan Vengadassalapathy, Vidhya Rekha Umapathy, Shazia Fathima Jaffer Hussain, Kalaiselvi Krishnamoorthy, Durairaj Sekar, Chella Perumal Palanisamy, Surapaneni Krishna Mohan, Ponnulakshmi Rajagopal

**Affiliations:** 1 Centre of Molecular Medicine and Diagnostics (COMManD), Department of Biochemistry, Saveetha Dental College & Hospital, Saveetha Institute of Medical & Technical Sciences, Chennai 600077, India; monishapandu3@gmail.com (M.P.); susi.sumathi98@gmail.com (K.K.); 2 Department of Basic Medical Sciences, College of Medicine, University of Sharjah, Sharjah 27272, United Arab Emirates; meladl@sharjah.ac.ae; 3 Department of Basic Medical Sciences, College of Medicine, AlMaarefa University, Riyadh 71666, Saudi Arabia; msharbini@mcst.edu.sa (M.E.-S.); mabdulrahman@mcst.edu.sa (M.A.E.A.); 4 Department of Pharmacology, Saveetha Medical College and Hospital, Saveetha Institute of Medical & Technical Sciences, Chennai 602105, India; srinivasanv.smc@saveetha.com; 5 Department of Public Health Dentistry, Sree Balaji Dental College and Hospital, Pallikaranai, Chennai 600100, India; drvidhyarekha@gmail.com; 6 Department of Oral and Maxillofacial Pathology, Ragas Dental College and Hospitals, Chennai 600119, India; shaziafathimarizwan@gmail.com; 7 Cellular and Molecular Research Centre, Saveetha Dental College & Hospitals, Saveetha Institute of Medical & Technical Sciences, Saveetha University, Chennai 600077, India; durairajsekar.sdc@saveetha.com; 8 State Key Laboratory of Biobased Material and Green Papermaking, College of Food Science and Engineering, Qilu University of Technology, Shandong Academy of Science, Jinan 250353, China; perumalbioinfo@gmail.com; 9 Departments of Biochemistry, Molecular Virology, Research, Clinical Skills & Simulation, Panimalar Medical College Hospital & Research Institute, Varadharajapuram, Poonamallee, Chennai 600123, India; krishnamohan.surapaneni@gmail.com; 10 Department of Central Research Laboratory, Meenakshi Ammal Dental College and Hospitals, Chennai 600095, India

**Keywords:** phytosterols, insulin resistance, diabetes mellitus, obesity, cholesterol, therapeutic implications

## Abstract

Natural products in the form of functional foods have become increasingly popular due to their protective effects against life-threatening diseases, low risk of adverse effects, affordability, and accessibility. Plant components such as phytosterol, in particular, have drawn a lot of press recently due to a link between their consumption and a modest incidence of global problems, such as Type 2 Diabetes mellitus (T2DM), cancer, and cardiovascular disease. In the management of diet-related metabolic diseases, such as T2DM and cardiovascular disorders, these plant-based functional foods and nutritional supplements have unquestionably led the market in terms of cost-effectiveness, therapeutic efficacy, and safety. Diabetes mellitus is a metabolic disorder categoriszed by high blood sugar and insulin resistance, which influence major metabolic organs, such as the liver, adipose tissue, and skeletal muscle. These chronic hyperglycemia fallouts result in decreased glucose consumption by body cells, increased fat mobilisation from fat storage cells, and protein depletion in human tissues, keeping the tissues in a state of crisis. In addition, functional foods such as phytosterols improve the body’s healing process from these crises by promoting a proper physiological metabolism and cellular activities. They are plant-derived steroid molecules having structure and function similar to cholesterol, which is found in vegetables, grains, nuts, olive oil, wood pulp, legumes, cereals, and leaves, and are abundant in nature, along with phytosterol derivatives. The most copious phytosterols seen in the human diet are sitosterol, stigmasterol, and campesterol, which can be found in free form, as fatty acid/cinnamic acid esters or as glycosides processed by pancreatic enzymes. Accumulating evidence reveals that phytosterols and diets enriched with them can control glucose and lipid metabolism, as well as insulin resistance. Despite this, few studies on the advantages of sterol control in diabetes care have been published. As a basis, the primary objective of this review is to convey extensive updated information on the possibility of managing diabetes and associated complications with sterol-rich foods in molecular aspects.

## 1. Introduction

Diabetes mellitus is becoming more common around the world. Diabetes affects approximately 537 million adults (20–79 years) as of 2021. By 2030, the overall number of diabetics is expected to reach 643 million, and by 2045, it will reach 783 million. Three out of four adults with diabetes live in a low- or middle-income country. India has the second-largest diabetes population (about 75 million) [1]. One of the major health issues in the modern world is the rising prevalence of type-2 diabetes. Obesity and diabetes have become more prevalent globally as a result of changes in lifestyle, such as high-calorie diets and sedentary behaviour [2]. T2DM is now being managed with insulin and pharmaceutical treatments. The current treatments for T2DM are unable to cope with the disease’s diverse manifestations, thus prompting the creation of an alternative therapy that is effective in cost and in reversing the disease. Plants have offered a useful supply of numerous natural goods, as well as medicinal treatments and pharmaceuticals, for thousands of years [3]. Phytomedicines are now utilised by approximately 80% of the world’s population to prevent and treat an extensive range of health problems, according to the World Health Organization (WHO) [4,5]. Natural active ingredients in medicinal herbs, plants, vegetables, and fruits are useful in the control and cure of a wide range of human ailments, including cancer, cardiovascular disease, diabetes, obesity, metabolic syndromes, and neurological issues. Sterols, in particular, play a central role in the growth of cell and organelle membranes in higher animals. In the animal kingdom, they are identified as cholesterol, whereas in the plant kingdom, they are recognised as phytosterols [6].

**Figure 1:**
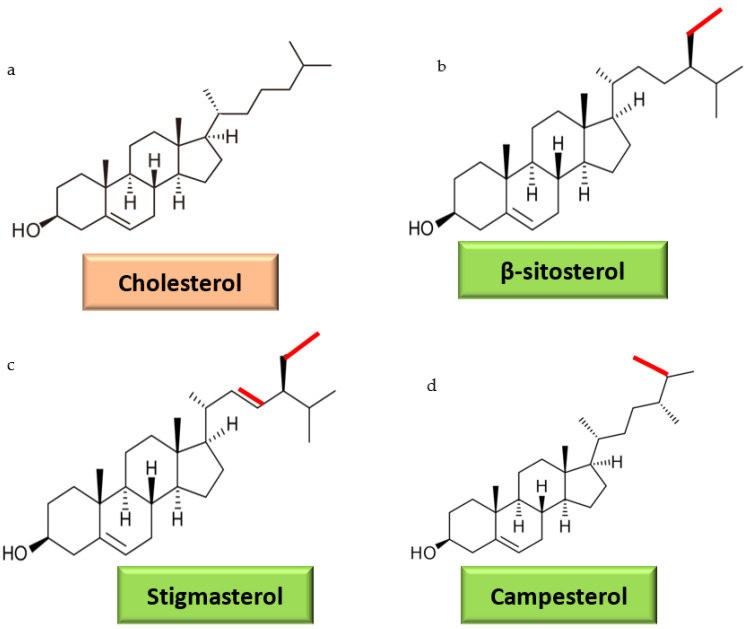
(**a**) Structure of cholesterol, (**b**) structure of β-sitosterol (ethyl group represents in red line), (**c**) structure of stigmasterol (ethyl group and double bond represented by red line), and (**d**) structure of campesterol (methyl group represented by red line).

Phytosterols are phytonutrients that resemble cholesterol in structure and action. Thus far, more than 250 phytosterols and 4000 triterpenes have been reported. The presence of an ethyl or methyl group on the 24th carbon is used to differentiate phytosterols from cholesterol and designate them as 24methylsterols (campesterol) or 24ethyl sterols (sitosterol and stigmasterol) (Figure 1). Some of the types found in plants are steryl glycosides, esterified or free fatty acids, and acylated glycosides [7]. Plant membrane sterols, such as stigmasterol, β-sitosterol, and campesterol, in higher concentrations can help lower blood cholesterol levels and thus prevent metabolic disorders. Phytosterols are abundant in cereals, vegetables, fruits, and nuts, and a daily dose of 2 g decreases cholesterol absorption by 30–40% and LDL cholesterol by 10% on average [8].

Phytosterol has been studied extensively for the treatment and management of diabetes, a variety of malignancies, cardiovascular disorders, atherosclerosis, and skin problems [9,10]. These phytosterols have been shown to have multiple therapeutic implications, such as hypolipidemic, anti-inflammatory, antioxidant, antiproliferative, and hypoglycemic potential through experimental evidence (Table 1). In recent years, a rising number of anti-diabetic studies have demonstrated phytosterols to be helpful in vivo and in vitro, and it is becoming more popular as a natural treatment. However, a comprehensive study of the preventative and prospective mechanisms of food-derived phytosterols for diabetes and its consequences has yet to be identified. The significance of phytosterols in the treatment of T2DM and its implications, as well as the theoretical potential for long-term pharmacological research and clinical diabetic treatment, are the focus of this review.

## 2. Insulin Pathophysiology

Insulin resistance occurs when insulin is ineffective in getting plasma glucose to enter a body’s cells and be used for energy by the cells, even when there is enough insulin in the blood. The cells, in other words, are resistant to insulin. Furthermore, even when glucose is not required, the liver may continue to release glucose into the bloodstream. Insulin resistance in peripheral target tissues, as well as reduced insulin secretory capacity of pancreatic β-cells, play a role in the development of type 2 diabetes [22]. The pancreatic islets in most type 2 diabetes patients keep cells in proportion to cells that are not even altered on a regular basis, and normal cell mass appears to be sustained [23]. Pancreatic islet amyloid, which is induced by amylin deposition, affects a significant number of type 2 diabetics [24]. The delayed and muted response to glucose, as well as abnormalities in insulin secretion, are early symptoms of disrupted β-cell function. Increased free fatty acids in serum may have a role in β-cell failure, according to a few studies [25].

Insulin resistance can be induced by a number of factors. The most prominent reasons include increased production of reactive oxidative species (ROS), impaired glucose and fatty acid oxidation, Endoplasmic Reticulum (ER) stress, and mitochondrial malfunction. Insulin resistance can also be induced by a lack of exercise, obesity, or chronically high blood sugar levels in sensitive people, all of which can predispose them to certain genes. Genetic variables appear to be the most important determinants of type 2 diabetes mellitus development [26]. Genetically determined intracellular post-receptor abnormalities are most likely to blame. Obesity, hypertension, hyperlipidemia, and coronary artery disease are all disorders that can be caused by hyperinsulinemia [27]. Patients usually lose their early insulin secretory response to glucose and may release a lot of proinsulin [28]. Despite the fact that fasting plasma insulin levels in type 2 diabetic individuals may be normal or even higher, glucose-induced insulin production is reduced. When insulin levels are low, insulin-mediated glucose absorption is reduced but does not prevent hepatic glucose synthesis [29]. As a result, the cell’s response to insulin signalling may be altered, and GLUT4 expression may decrease. As a consequence, blood glucose levels remain high, and the pancreas responds by secreting more insulin, resulting in a positive feedback loop that raises intra-vascular insulin levels while desensitising peripheral tissues to insulin. The biomolecular consequence of this cycle is that GLUT4 expression in the cell membrane continues to be downregulated, worsening the issue [30,31,32,33]. Hyperglycemia, hyperinsulinemia, and increased oxidative stress in tissues are all systemic consequences of this dysfunction.

**Figure 2:**
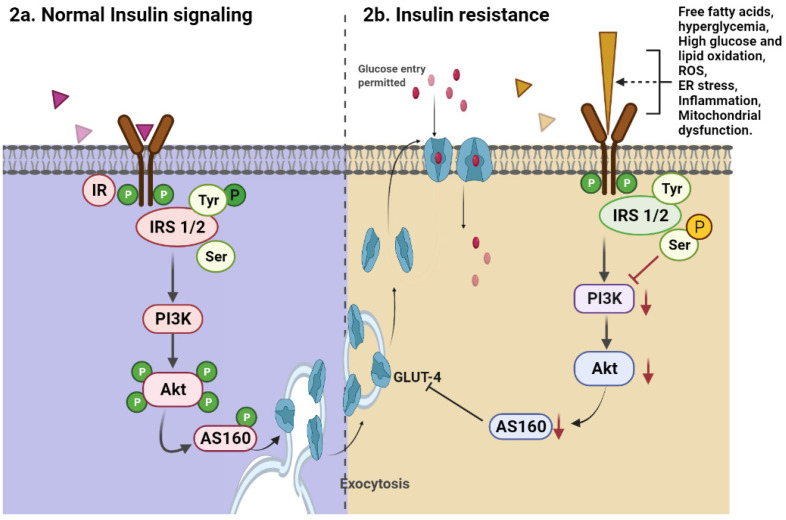
Molecular mechanism of action of insulin signalling under physiology and pathophysiological condition. Normal insulin signalling begins with insulin binding to the insulin receptor, then phosphorylation of the Tyr residue of IRS 1/2, and finally, activation of the PI3K and Akt enzymes (**a**). Activated Akt causes glucose translocation for oxidation by activating the GLUT4 transloaction proteins. In insulin resistance, instead of Tyr phosphorlation, serine residue phosphorlation after insulin binding to the insulin receptor leads to decreased activation of PI3K and Akt, resulting in decreased GLUTt4 translocation and glucose uptake. The red arrow indiactes IRS1/2 serine phosphorylation-mediated inhibition PI3K/Akt/AS160-mediated signalling that leads to insulin resistance due to various factors (**b**).

According to recent data and our findings, insulin resistance, which can be caused by a variety of factors, such as hyperglycemia, obesity, free fatty acids, reactive oxygen species (ROS), and infection, regulates the above-mentioned insulin signalling (Figure 2a) and causes changes in a variety of genes and protein expression. A high-fat diet, for example, alters glucose and fat oxidation in the body, resulting in the production of reactive oxygen species (ROS). Inflammatory cytokines, such as tumor necrosis factor-alpha (TNF-α), interleukin-1 (IL-1), and interleukin-6 (IL-6), as well as alterations in insulin signalling genes, are created as a result. Furthermore, the insulin signalling cascade is disturbed, which disrupts glucose translocations, resulting in reduced glucose uptake and insulin resistance [15,16,17]. Animal models of diabetes and cell lines will be beneficial in the search for factors associated with the formation of a defective plasma insulin pattern in diabetes, as well as in understanding the mechanisms involved [25,29,34,35]. Such research could lead to the discovery of specific variables involved in the pathogenic process, paving the way for new pharmaceutical interventions aimed at restoring normal plasma insulin levels. The pathogenesis of insulin resistance is depicted in Figure 2b.

## 3. Dietary Sterols Overview

**Figure 3:**
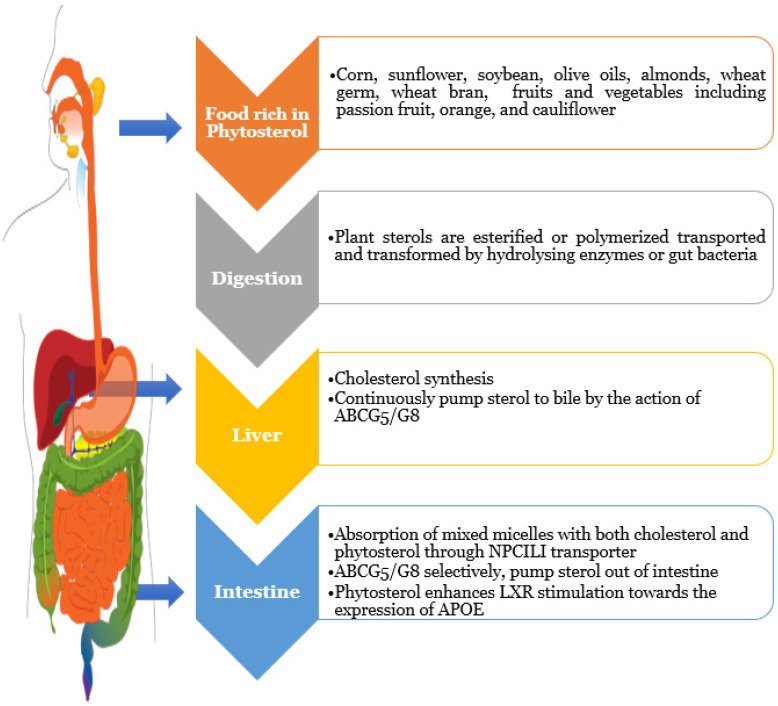
Phytosterols metabolism towards the regulation of cholesterol absorption. When phytosterol-rich foods are consumed, they are processed by liver enzymes that are comparable to those used to break down choletserol. It will reach the enterocytes after digestion, where phytosterol metabolism differs from cholesterol metabolism. Because these phytosterols are poor substrates of acyl-coA cholesterol actytransferase, they are not esterified and remain in the free form within the cell. Finally, the ATP-driven transporter heterodimer ABCG5/8 excretes the bulk of the free plant sterols back into the interstinal lumen.

Plants produce two forms of phytosterols: plant sterols and plant stanols. Research studies have associated the consumption of phytosterols (2 g/day) in the form of poly- and monounsaturated fatty acids (PUFA and MUFA) with a significant reduction (8–10%) in LDL-cholesterol (Figure 3). Phytosterols can be set up in corn, sunflower, soybean, and olive oils, as well as oleaginous fruits, including wheat germ, wheat bran, almonds, cauliflower, passion fruit, and oranges [35,36,37]. Only around 0.5–2% of plant sterols and 0.04–0.2% of plant stanols are absorbed when plant sterols- and stanols-rich meals are consumed. Consuming 18 g of plant sterols per day, on the other hand, reduces blood cholesterol in humans. Plant sterols absorb in the gut significantly more slowly than cholesterol, which is largely found in the liver and eliminated in the bile (1200–1500 mg/day). The liver’s cytochromes and enzymes are essential because some plant sterols can be absorbed and transported in their natural state. Hydrolysing enzymes or gut bacteria must transport and modify the most of plant sterols because they are esterified, polymerised, or contain glycosyl moieties [38].

In mixed micelles, phytosterols/stanols fight for solubilisation, lowering cholesterol absorption via the Niemann-Pick C1-Like 1 (NPC1L1) transporter. The ATP-binding cassette sub-family G member 5 (ABCG5/G8) transporter regulates its absorption in the colon and liver, which is lower than cholesterol absorption [39]. Plant sterol levels almost normalised after a liver transplant with sitosterolemia who had a hereditary abnormality in the gut [40]. When fasting, ABCG5 and ABCG8 may preferentially pump sterols out of intestinal cells as a first-line defence against food intake, but the liver may continuously pump sterols into bile and therefore sustain a low non-cholesterol sterol level in the circulation. By continuing to pump sterols when fasting, which are likely supplied to intestinal enterocytes via the high-density lipoprotein route, the gut may be able to diminish whole-body sterol pools [41]. Plant sterols can also stimulate LXR receptors, which control apolipoprotein E (APOE) production and are largely catalysed by microsomal cytochromes. When LXR receptors are activated, the activity of ABCG5/G8 transporters increases, increasing phytosterol absorption. The SR-IB receptors on the cell surface of the liver and adrenal glands capture the exported high density lipoprotein-like particles by the integrated pytosterols. This receptor is required for HDL cholesterol absorption, as well as, more importantly, lipid and cholesterol metabolism in the brain, where HDL serves as the principal source of lipids and cholesterol [42]. While enterocytes and hepatocytes have ABCG5/G8 plant sterol transporters, they are not present in other cell types. Plant sterol absorption is linked to lipoprotein uptake and coupled with cholesterol uptake regulatory mechanisms because endogenous production of plant sterols is not controlled in humans. As a result, as a way of excreting plant sterols from tissues into the circulation, a cell oxidises plant sterols to generate oxyphytosterols [43]. The biliary system eliminates phytosterol, which appears to be quicker than cholesterol. The endogenous phytosterol pool is tiny in contrast to cholesterol because of minimal absorption in the gut and quick excretion through bile [44,45].

## 4. Therapeutic Functions of phytosterols

### 4.1. Reduces Intestinal Glucose Absorption

Chronic hyperglycemia promotes the generation of free radicals, which can lead to lipid peroxidation, non-enzymatic protein glycation, structural and functional changes in the basement membrane and collagen, and enzyme inactivation, all of which contribute to complications of diabetes [46,47]. Glycemic management is one of the most important aims for diabetes treatment since oxidative stress-induced hyperglycemia plays such a big role in the progression of diabetes. As a consequence, researchers are growing more interested in understanding both crude and separated herbal components, as well as exploiting possible phytosterols for medical purposes in lowering diabetes’ adverse effects [48]. Dietary pytosterols and stanols, which can be found in our food and functional foods, are well-known for their anti-hyperglycemic properties, which help manage diabetes. According to research, an extract of *Artocarpus heterophyllus* (jackfruit), a prominent ingredient in traditional Indian cuisine and abundantly available in India and its neighbours, improves glucose tolerance in both healthy and diabetic people. Its leaves are hypoglycemic and hypolipidemic, making them potentially valuable for a diabetes cure in the future. Sapogenins, cycloartenone, cycloartenol, β-sitosterol, and tannins are seen in the stem and leaves of the plant [49,50,51].

The hyperglycemic phenotype of diabetic KK-Ay mice, a cross of black KK females with obese yellow Ay males, which are hyperglycemic, insulin resistant, and obese, was significantly alleviated after 4 weeks of stigmasterol (SMR) treatment, with significantly lower fasting glucose, demonstrating a notable hypoglycemic effect against T2DM. The body weight and fasting blood glucose (FBG) levels reduced after SMR treatment, the blood insulin concentration dropped dramatically, and the oral glucose tolerence test (OGTT) demonstrated that phytosterol improved insulin sensitivity [20]. β-sitosterol is a strong neuroprotective, chemoprotective, and anti-diabetic natural sterol that increases the generation of total antioxidants in cells. We previously reported that β-sitosterol had beneficial effects by lowering plasma glucose levels through one of two mechanisms or a combination of both, namely a decrease in gut glucose absorption or an increase in the glycogenic and glycolytic pathways with a corresponding reduction in the gluconeogenesis and glycogenolysis pathways. This might affect glucose metabolism [16]. Similarly, multiple studies have shown that β-sitosterol has anti-diabetic properties [52,53].

### 4.2. Reduces Intestinal Lipid Absorption

Natural plant components such as phytosterol have received a great deal of press recently because of the link between their consumption and a lower risk of diabetes, cardiovascular disease, and cancer. For the first time, in 1953, phytosterols were identified to assist patients in lowering their cholesterol levels [54,55]. Phytosterols, also known as stanols, have been found to reduce total and LDL cholesterol levels in adults. Although they have a wide range of commercial applications, guava seeds (*Psidium guajava*) are discarded by the beverage and juice industries. Using chromatographic techniques, the phytochemicals and free-radical scavenging activities of Guava Seed Oil (GSO) n-hexane extract, as well as alterations in serum lipids and fatty acid profiles in rats, were investigated. In cell cultures, GSO has been reported to produce phenolic compounds (such as chlorogenic acid and its derivatives), as well as phytosterols (such as stimasterol, β-sitosterol, and campesterol), and to have a concentration-dependent radical-scavenging action. GSO showed no impact on blood fatty acid levels in rats over time, but it did affect cholesterol and triglyceride levels in a concentration-dependent manner [56,57].

Total sterol concentrations in vegetable oils ranged from 272.3 mg/kg in coconut oil to 6169.7 mg/kg in evening primrose oil, with the highest concentrations of β-sitosterol and the largest phytosterol amount in evening primrose oil [58]. After obtaining a baseline lipid profile, the therapeutic benefits of methanol extracts of *Moringa oleifera* seed (MOSE) at 100 and 200 mg/kg bw of rats were investigated for 6 weeks. When compared to the regular orlistat, all five groups had a decrease in VLDLc cholesterol. There was a considerable increase in HDL cholesterol in the group administered 200 mg/kg MOSE (HDLc). On the other hand, the group given 100 mg/kg MOSE saw a potentially significant increase in atherogenic index (AI). The structure–activity relationship between the isolated component and its derivative equivalent was discovered, and the methanol extract of *Moringa oleifera* seed was found to impact the lipid profile [59]. The anti-hyperglycemic and anti-hyperlipidemic activities of an ethanolic extract of *Glycyrrhiza glabra (licorice)* roots were compared to the standard drugs metformin and glimepiride in streptozotocin(STZ)-induced diabetic rats, and all lipidemic indices were significantly reduced, including VLDL-C, LDL-C, total cholesterol (TC), and triglycerides (TG). The anti-cholesterol properties of a 70% (*v*/*v*) ethanol extract of *Abelmoschus esculentus* L. (okra fruit) and its nanoparticles were also tested in vivo. The zeta potential of the nanoparticles used was −26.72, and the particle size was 134.7 nm. Total cholesterol levels reduced by 48.68%, 32.44%, and 42.95% in the positive control, extract, and nanoparticles groups, respectively [60].

**Figure 4:**
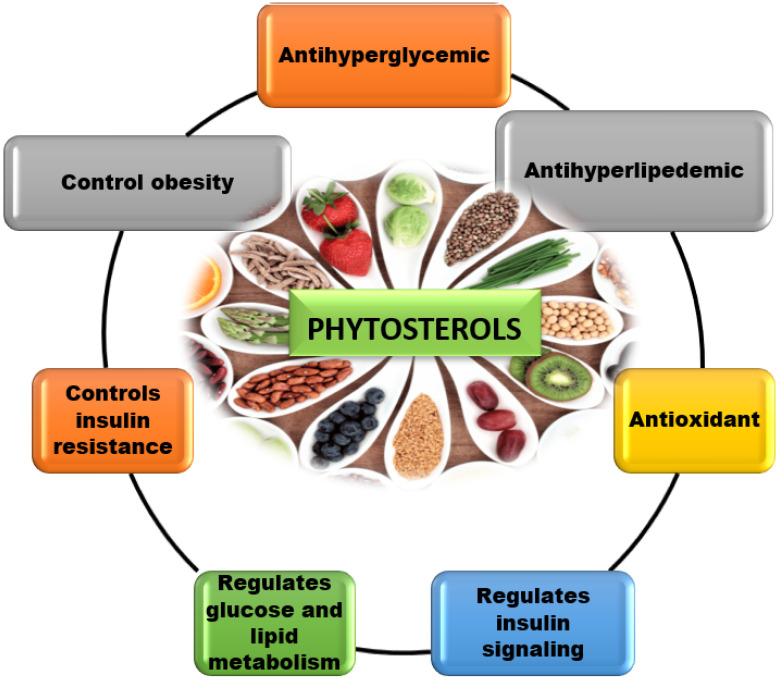
Overall therapeutic action of phytosterol. Phytosterol resembles cholesterol, which has been proven to inhibit interstinal cholesterol absorbtion and elevates antioxidants, making it a more effective antidiabetic, hypolipidemic, and anti-inflammatory agent. In addition to these actions, active phytosterol regulates insulin resistance, glucose and lipid metabolism, insulin signalling, and obesity management.

Higher levels of β-sitosterol and campesterol in the blood are linked to lower cholesterol absorption [61]. In the intestines, phytosterol intake reduces cholesterol absorption. Phytosterols containing β-sitosterol have been proven to reduce dietary cholesterol absorption in the intestine in various studies. β-sitosterol prevents cholesterol absorption through the intestine, inhibiting hepatic cholesterol synthesis pathways and lowering blood cholesterol [62]. Ingestion of plant materials high in β-sitosterol lowers total blood cholesterol levels in experimental animals, as expected [63]. Angela Oliveira Godoy Ilha et al. [64] studied the effects of phytosterol on biomarkers involved in atherosclerosis progression and whether these effects are independent of changes in plasma LDL-c levels in 38 moderately hypercholesterolemic volunteers who were randomly assigned to consume 400 mL/day of soy milk or soy milk with phytosterol (1.6 g/day) for four weeks in a double-blind, placebo-controlled, cross-over study. Phytosterol therapy lowered endothelin-1 plasma concentration by 11% regardless of fluctuations in LDL-c plasma levels, according to the findings. There were no differences in IL-6, SAA, fibrinogen, hs-CRP, TNF, or VCAM-1 between the placebo- and phytosterol-treated groups. Phytosterol considerably lowered total plasma cholesterol, LDL-c, triglycerides, and apo B levels without affecting HDL-c levels, adding to our knowledge of the role of phytosterols in the prevention of cardiovascular and endothelial dysfunction (Figure 4).

### 4.3. Alters Insulin Resistance in Tissues and Organs

T2DM is caused by two mechanisms: (1) glucose, a main metabolic fuel produced in the liver through the gluconeogenesis pathway during fasting when insulin is downregulated, is a key contributor to hyperglycemia; (2) reduced insulin-mediated glucose absorption and resultant diabetic organ damage are caused by insulin resistance in peripheral target tissues and altered insulin secretory capacity of pancreatic β-cells [56]. Insulin resistance is thus restricted to the liver and other peripheral organs. Peripheral insulin resistance, for example, is characterised by reduced insulin-mediated glucose uptake and utilisation in muscle and adipose tissue but enhanced fat breakdown in adipose tissue. Insufficient gluconeogenesis inhibition, decreased glycogen synthesis, and increased lipid buildup characterise selective hepatic insulin resistance [65]. According to recent research, this causes peripheral insulin resistance, which leads to a decrease in glucose uptake from the circulation as a result of an increase in incoming glucose conversion to the liver. Furthermore, phytosterols, rather than the normal C24 bile acids, can be transformed to C21 bile acids in the livers of mammals. Phytosterols may offer health benefits in both animals and people, including lowering cholesterol, reducing the risk of diabetes and coronary heart disease, anti-inflammatory effects, activating apoptosis in cancer cells, and disease prevention and treatment [66].

Insulin sensitivity varies according to the target tissue. Skeletal muscle is more insulin-sensitive than liver and adipose tissue. For example, insulin resistance in skeletal muscle often precedes insulin resistance in the liver and adipose tissue in the early stages of T2DM, and it can also include abnormal metabolism in other tissues. Because IR contact and activation regulate physiological insulin signalling, which causes structural changes and autophosphorylation, culminating in the recruitment and phosphorylation of intracellular proteins such as insulin receptor substrates (IRS) and Sh2-containing collagen-related protein (Shc). IRS proteins stimulate the PI3K-Akt pathway, while Shc activates Ras-MAPK pathway. Phosphoinositol 3-kinase (PI3K) is activated by IRS proteins, which results in the synthesis of phosphatidylinositol 3,4,5-triphosphate (PIP3), a second messenger that recruits and stimulates 3-phosphoinositide-dependent protein kinase-1 (PDK-1). When PDK-1 is triggered, it phosphorylates Akt, which regulates glucose uptake by permitting the insulin-sensitive glucose transporter GLUT4 to translocate to muscle and fat cell membranes. Meanwhile, Akt has a role in lipid synthesis, gluconeogenesis, glycogen synthesis, and other insulin-related metabolic activities [67]. Insulin signalling is disturbed at multiple levels during insulin resistance, resulting in decreased glucose absorption in insulin-sensitive peripheral tissues, according to our earlier findings on the liver and peripheral organs. Insulin, insulin-like growth factors, and a variety of other growth factors are all members of the ligand-activated receptor family, which also includes the insulin receptor. It’s a tyrosine kinase-family transmembrane signalling protein. The antioxidant and hypolipidemic actions of phytosterol in the liver, adipose tissue, and skeletal muscle may explain why β-sitosterol therapy restored IR mRNA expression while partially lowering lipid levels in diabetic rats [16,17].

**Figure 5:**
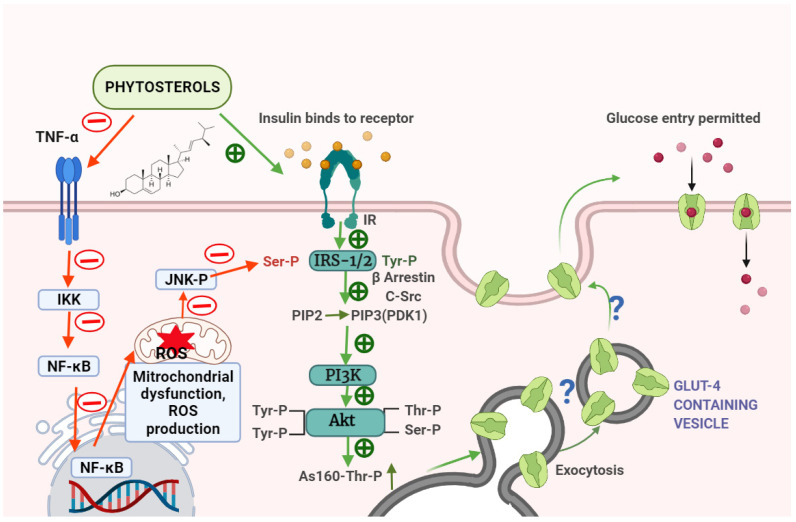
Schematic mechanism of plant sterols regulating insulin signalling and inflammation. The mechanism of action of phytosterol is illustrated in the figure based on the literature available. As phytosterol is a potent antidiabetic, it enhances insulin signalling molecules, such as IR, IRS 1/2, PI3K, Akt, and AS160, which promotes GLUT4 synthesis and glucose absorption. There is still a lack of research on the effects of phytosterols on the GLUT4 translocation pathway (denoted as ?). Phytosterol also suppresses inflammatory events by inhibiting the IKK/NF-kB and c-Jun-N-terminal kinase (JNK) signalling pathways. Plant sterol-facilitated activation is indicated by a green plus symbol. The red circle denotes phytosterol’s inhibitory effect on bothinsulin signalling and inflammation.

GLUT4 is one of 13 glucose transporter proteins found in adipose, skeletal, and cardiac muscle cells. GLUT4 translocation and expression levels are related to whole-body insulin-mediated glucose homeostasis. In vitro and in vivo studies revealed a significant alteration of stigmasterol’s anti-diabetic action and a possible mechanism. SMR boosted GLUT4 translocation in L6 cells by 1.44-fold. Different dosages of phytosterol were administered to L6 cells, which had a significant impact on glucose uptake. Oral administration of SMR to KK-Ay mice enhanced insulin resistance and oral glucose tolerance by lowering fasting blood glucose levels and blood lipid markers, such as triglycerides and cholesterol. GLUT4 expression was increased in L6 cells, skeletal muscle, and white adipose tissue [20]. These results imply that phytosterols improve glucose management in type 2 diabetes patients’ liver and peripheral organs through activating IR and GLUT4 (Figure 5).

### 4.4. Antidiabetic Activity of Phytosterol Derivatives

Saponins are made up of an aglycone unit that is attached to one or more carbohydrate chains. A sterol or the more frequent triterpene unit makes up the aglycone or sapogenin unit. These glycosidic molecules, known as sterol saponins, have been demonstrated to offer potential therapeutic effects and are being considered as an alternative to insulin in diabetic patients. The role of saponin extracted from the root bark of Berberis vulgaris linn was examined. In male Wistar rats, saponins extract (25 mg/kg) therapy began ten days after STZ injection and lasted three weeks (190–230 g). When compared to the diabetic control group, the diabetic group treated with barberry saponin extract had highest hypoglycemic activity (73.1 on day 1 and 76.03% on day 21). The hypoglycemic impact of saponins identified in Berberis vulgaris linn’s root bark is assumed to be due to saponins, which may stimulate surviving beta cells. Saponin extracts improved lipid profiles while also having a hypoglycemic effect, indicating that they might be effective in the treatment of diabetes [68]. Alloxan-induced diabetic male Wistar albino rats (200 g to 250 g) were administered saponin derived from *Cochlosperum vitifolium* orally at doses of 200, 400, and 600 mg/kg body weight daily for 7 days in another investigation (200 g to 250 g). After 3 and 7 days of therapy, the saponin extract reduced blood glucose levels by 35.98% to the metformin group. For diabetic rats’ livers to achieve normal homeostasis, the saponin extract’s capacity to decrease elevated blood glucose levels to normal levels is crucial [69].

Diosgenin, a natural steroidal sapogenin, is abundant in plants such as fenugreek, yam, and *Polygonatum verticillatum*. Diosgenin has been studied extensively for cancer management and therapy [62]; cardiovascular illnesses, including atherosclerosis [70]; diabetes mellitus [71]; and skin diseases [72]. It also has a variety of biological properties, including hypolipidemic, anti-inflammatory, anti-proliferative, and hypoglycemic activity, and as a powerful anti-oxidant, it has a positive impact on diabetes and its consequences across various targets and pathways. Diosgenin, which makes up the majority of the plant, is the active component in fenugreek. It has been identified as a key regulator of fenugreek’s biological activities in studies looking at its potential as a diabetes therapy [73,74,75].

## 5. Conclusions and Prospective Studies

Diabetes mellitus and its accompanying disorders have become more prevalent all over the world, and they are connected to dietary patterns either directly or indirectly. Dietary plant sterols and stanols, which are included in our diet and functional foods, have long been recognised to limit intestinal cholesterol absorption, resulting in lower levels of LDL cholesterol. According to recent research, plant sterols and stanols appear to have a slew of other health benefits, including the ability to manage diabetes and its consequences. Phytosterols are key micronutrients that affect glucose and lipid metabolism, modifying insulin resistance. They are structurally and functionally comparable to cholesterols. There is still a shortage of literature on the mechanism of phytosterol acton in glucose absorption and GLUT4 transloaction. If this gap is closed in the future, any action of phytosterol that increases glucose absorption for oxidation and promotes the GLUT4 transloaction will be a potent phytotherpy for type 2 diabetes. More in vitro and in vivo models are needed to investigate the role of phytosterol in glucose uptake and oxidation, as well as the controlling influence of plant sterols on insulin resistance and inflammation. As a result, phytosterols and their derivatives have a wide range of biological actions that benefit human and animal health, and their widespread usage should be encouraged to minimise diabetes-related illnesses.

## Figures and Tables

**Table 1:** Studies on biological activity of phytosterol from plants or as compounds.

S. No.	Phytosteroor Its Sources	Model	Type of Study	Biological Activity of Phytosterol	Implication	Reference
1	0.4% stigmasterol and β-sitosterol in the diet	Mice fed a Western-style high-fat diet	In vivo	Antilipidemic activity	The study found that phytosterols were beneficial in preventing nonalcoholic fatty liver disease (NAFLD) caused by a high-fat Western diet (HFWD) (NAFLD). In this long-term (33-week) investigation, phytosterols, at a dose comparable to that advised for the alleviation of NAFLD, were predominantly related to the decreases in hepatic cholesterol, triglycerides with polyunsaturated fatty acids, and modifications of hepatic-free fatty acid.	[11]
2	Dry leaves of *Eryngium foetidum* L. hexane extract	Six female Swiss mice weighing 25–30 g each	In vivo	Anti-inflammatory activity	In the chromatographic fractionation of the *Eryngium foetidum* L. leaves isolate, a-cholesterol, brassicasterol, campesterol, stigmasterol (as the main component, 95%), clerosterol, β-sitosterol, D5-avenasterol, D524-stigmastadienol, and D7-avenasterol were all detected. The topical anti-inflammatory effects of hexane extract and stigmasterol reduced oedema in the same proportion in both animal tests (acute and chronic). Both the extract and the compound greatly reduced myeloperoxidase activity in the acute phase but not in the chronic phase, suggesting that *Eryngium foetidum* L. leaves could be used to treat contact dermatitis. Because stigmasterol possesses anti-inflammatory activities on the epidermis but is not a major anti-inflammatory agent, other bioactive components are likely involved in the hexane extract’s efficacy.	[12]
3	*Lagerstroemia speciosa* seed ethanolic extract		In silico	Breast cancer	The ADME (Adsorption, Distribution, Metabolism, Excretion) characteristics, pharmacokinetic features, drug-likeliness, and acute toxicity of the discovered phytosterols compounds are predicted by ethanolic extracts *Lagerstroemia speciosa* seeds GC–MS analysis and in silico analysis. GC–MS analysis identified 29 chemicals from the extract, four phytosterol derivatives, cholesterol margarate, 7-dehydrodiosgenin, stigmastan-3,5-diene, and γ-sitosterol, which were shown to be non-toxic, non-carcinogenic, and non-mutagenic. The extent of molecular interaction with breast cancer targets is also revealed by molecular docking studies. This research reveals that phytosterols derived from *Lagerstroemia speciosa*’s ethanolic seed extract could be a potential anti-breast cancer alternative.	[13]
4	β-sitosterol	Male albino wistar rats	In vivo	Anti-inflammatory activity	In high fat diet- and sucrose-induced type-2 diabetic rats, β-sitosterol treatment normalises raised serum levels of proinflammatory cytokines, such as leptin, resistin, tumour necrosis factor-(TNF-α), and interleukin-6 (IL-6) and increases anti-inflammatory adipocytokines, such as adiponectin. In diabetic rats’ adipocytes, β-sitosterol lowers sterol regulatory element binding protein-1c (SREBP-1c) and increases Peroxisome Proliferator–Activated Receptor-γ (PPAR-γ) gene expression. In β-sitosterol-treated groups, c-Jun-N-terminal kinase-1 (JNK1), inhibitor of nuclear factor kappa-B kinase subunit beta (IKK), and nuclear factor kappa B (NF-kB) gene and protein expression were likewise considerably reduced. This study reveals that SIT prevents obesity-induced insulin resistance by reducing inflammatory events in the adipose tissue via inhibiting the IKK/NF-kB and c-Jun-N-terminal kinase (JNK) signalling pathways.	[14]
5	β-sitosterol	Male albino wistar rats	In vivo	Antidiabetic activity	Treatment with β-sitosterol restored the altered levels of blood glucose, serum insulin, testosterone, lipid profile, oxidative stress indicators, antioxidant enzymes, insulin receptor (IR), and glucose transporter 4 (GLUT4) proteins in a high-fat diet and sucrose-induced diabetic rats. This research shows that β-sitosterol improves glycemic control in high-fat and sucrose-induced type-2 diabetic rats through activating IR and GLUT4. In addition, the results of in silico analysis match those of in vivo testing.	[15]
6	β-sitosterol	Male albino wistar rats	In vivo	Antidiabetic activity	When compared to high-fat diet and sucrose induced type-2 diabetic rats, β-sitosterol increased the mRNA expression of IR and post-receptor insulin signalling molecules such as IRS-1, β-arrestin-2, Akt, AS160, and GLUT4, as well as the levels of IRS-1, p-IRS1-1Tyr632, Akt, p-AktSer473/Thr308, AS160, and p-AS160Thr642. In this study, in silico analysis revealed that β-sitosterol has a higher binding affinity for β-arrestin-2, c-Src, and IRS-1, as well as Akt proteins, and has been shown to reduce insulin resistance, as evidenced by in vivo data. According to the findings, β-sitosterol reduces the potential effects of a high-fat diet on adipose tissue.	[16]
7	β-sitosterol	Male albino wistar rats	In vivo	Antidiabetic activity	In this study, high-fat diet and sucrose-induced diabetic rats showed reduced glucose and insulin tolerances, as well as insulin signalling molecules (IR and GLUT4) and glycogen levels. Serum insulin, lipid profile, LPO, H_2_O_2_, and OH* levels were shown to be higher in diabetic rats. The β-sitosterol therapy brought blood glucose, insulin, lipid profile, oxidative stress indicators, IR, and GLUT4 protein levels back to normal. This research suggests that IR and GLUT4 activation by β-sitosterol improves glycemic control in the gastrocnemius muscle of HFD-fed and sucrose-induced type 2 diabetic rats.	[17]
8	*Phormidium autumnale (P. autumnale)* cyanobacteria		In vitro	Neuroprotective properties	Compressed fluid technologies were used to produce phytosterol-rich extracts from *P. autumnale cyanobacteria* in order to investigate their potential neuroprotective capabilities in this work. The optimised compressed fluid extract demonstrated stronger in vitro neuroprotective efficacy than the non-enriched extract in bioactivity tests, such as acetylcholinesterase inhibition, lipoxygenase inhibition, and antioxidant capacity. The specificity of sterol interaction with acetylcholinesterase active sites was demonstrated using in-silico molecular docking investigations. These findings support future investigation of *P. autumnale* as a source of bioactive phytosterols, highlighting the utility of compressed fluid methods in obtaining phytosterol-rich extracts.	[18]
9	Soybean oil contains stigmasterol	KK-Ay mice	In vivo	Anti-diabetic activity	In L6 cells, stigmasterol greatly boosted GLUT4 translocation and glucose absorption. Furthermore, this in vivo research revealed that after 4 weeks of stigmasterol therapy, the hyperglycemic phenotype of diabetic KK-Ay mice was dramatically relieved, with significantly lower fasting glucose, indicating a considerable hypoglycemic impact against T2DM. In this investigation, stigmasterol appeared to offer potential benefits in the treatment of type 2 diabetes, with the likely method of targeting the GLUT4 glucose transporter, including increased GLUT4 translocation and expression.	[19]
10	Lophenol (Lo) and cycloartanol (Cy), minor phytosterols of *Aloe vera* gel	Zucker diabetic fatty (ZDF) rats	In vivo	Anti-diabetic activity	After 35 days of treatment, minor phytosterols from *Aloe vera* gel, lophenol (Lo) and cycloartanol (Cy), decreased hyperglycemia and random blood glucose levels in Zucker diabetic fatty (ZDF) rats, an obese animal model of type II diabetes. Apart from total cholesterol, continued treatment of Lo and Cy lowered blood free fatty acid (FFA) and triglyceride (TG) levels (T-Cho). Furthermore, with Lo (27.7%) and Cy (26.3%) treatment, the weights of total abdominal fat tissues in ZDF rats were considerably lower than in the control group. These findings imply that phytosterols obtained from *Aloe vera* may help to minimise visceral fat buildup and ameliorate hyperlipidemia and hyperglycemia.	[20]
11	Nuts	Human study	In vivo	Anti-diabetic activity	Randomised controlled studies of type 2 diabetes patients corroborated the positive benefits of nuts on blood lipids, which were also found in non-diabetic people, but no change in A1C or other glycated proteins was identified in the trials. However, acute feeding studies have shown that nuts can lower postprandial glycemia when consumed with a carbohydrate (bread). Additionally, nut intake was related to lower postprandial oxidative stress. Nuts have a favourable nutritional profile, being abundant in monounsaturated fatty acids (MUFA) and polyunsaturated fatty acids (PUFA), as well as being good sources of vegetable protein. Nuts may so increase the diet’s overall nutritious quality.	[21]

## Data Availability

The data presented in this study are available in this article.

## Sample Availability

Samples of the compounds are not available from the authors.
